# Tunable Energy Absorbing Property of Bilayer Amorphous Glass Foam via Dry Powder Printing

**DOI:** 10.3390/ma15249080

**Published:** 2022-12-19

**Authors:** Jungjin Park, John Howard, Avi Edery, Matthew DeMay, Norman Wereley

**Affiliations:** 1 Department of Aerospace Engineering, University of Maryland, College Park, MD 20742, USA; 2 Microsphere Material Solutions, Rockville, MD 20852, USA

**Keywords:** microspheres, cellular solid, dry printing, glass foam, energy absorption

## Abstract

The research in this paper entails the design of material systems with tunable energy-absorbing properties. Hollow glass microspheres of different densities are layered using dry powder printing and subsequently sintered to form a cellular structure. The tunability of the bilayer foams is investigated using various combinations of hollow microspheres with different densities and different thickness ratios of the layers. The mechanical responses to quasi-static uniaxial compression of the bilayer foams are also investigated. These bilayer samples show different mechanical responses from uniform samples with a distinctive two-step stress–strain profile that includes a first and second plateau stress. The strain where the second plateau starts can be tuned by adjusting the thickness ratio of the two layers. The resulting tunable stress–strain profile demonstrates tunable energy absorption. The tunability is found to be more significant if the density values of each layer differ largely. For comparison, bilayer samples are fabricated using epoxy at the interface instead of a sintering process and a different mechanical response is shown from a sintered sample with the different stress–strain profile. Designing the layered foams allows tuning of the stress–strain profile, enabling desired energy-absorbing properties which are critical in diverse impact conditions.

## 1. Introduction

All crash-worthiness applications, from occupant protection systems in automobiles or aircraft to electronic packaging, would benefit from the development of a material system with tunable energy absorption such that the stroking load is gradually increased, reducing the acceleration and peak stress experienced [1,2,3]. For example, adaptable passenger seats can be designed to accommodate passengers with different weights so that the transmitted stress can be efficiently absorbed via an adaptive energy absorbing layer [4].

Tunable energy absorption has been demonstrated through various mechanisms. These include architected lattices with jamming phase, soft mechanical meta materials with unusual swelling behavior, stimuli-responsive polymer materials, and carbon nano-tube arrays [5,6,7,8,9,10]. These concepts often consist of soft materials, such as hydrogels, and require external stimuli, such as magnetic field, mechanical stress, and temperature, to activate the tunable mechanical properties. Cellular structured materials are greatly used for energy absorbing applications [11,12,13,14]. Several research studies showed that graded cellular structures enabled gradual collapse leading to more energy absorption and this is beneficial for protecting payloads from dynamic loads [15,16,17,18]. Graded porous structures and syntactic foams are fabricated via gluing multi-layers or blowing agents. Recently, sintered glass foam with density gradients has exhibited progressive structural collapse during crush to demonstrate tunable energy absorption [5,19,20,21,22,23,24]. We previously showed the potential tunability of the glass foam via forming a bilayer structure that eventually controls the shape of the stress–strain curves in a certain level [24]. In this paper, we further demonstrate such tunability by employing layered foam structures utilizing different layer-wise foam densities.

Hollow glass microspheres (HGMs) have gained great attention for energy absorption applications because of their low density and high strength [12,25,26,27,28,29]. They are popularly used in syntactic foam which is a cellular polymeric matrix filled with hollow microspheres [30,31,32]. Recently, a cellular structure was successfully fabricated by sintering hollow glass microspheres [12,24,33,34,35,36]. A key advantage of HGM-based foams is that the cellular solid exhibits thermal stability up to 600 °C, which usually cannot be achieved with syntactic foam due to polymeric binders or matrices. Unlike syntactic foam, the glass foam manufacturing process usually does not require any matrix resin and other chemical additives [37,38,39].

**Figure 1:**
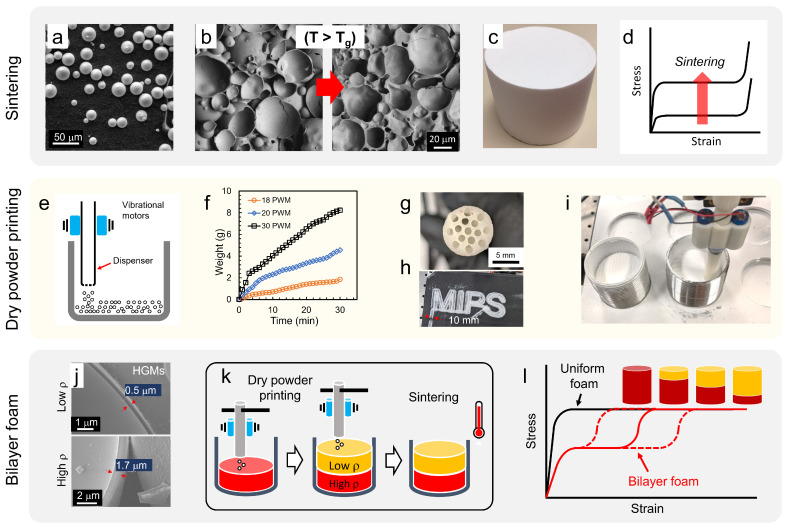
(**a**) SEM micrograph of hollow glass microspheres (HGM). (**b**) SEM micrographs of the amorphous glass foam microstructures for different durations thermal processing showing different levels of sintering. (**c**) Photograph of a sintered 4 inch diameter glass foam (**d**) Qualitative depiction of the stress–strain characteristics of sintering of glass foams (**e**) Schematic depiction of dry powder printing. (**f**) Dispensing rate of HGMs with different PWM (pulsed width modulation) via weight measurement as a function of dispensing time. (**g**) Bottom view of dispenser (**h**) Dry printed HGMs into letter MIPS. (**i**) Image of dry printing process. (**j**) SEM images of low and high density hollow glass spheres with wall thickness. (**k**) Schematic depiction of dry powder printing and bilayer glass foam sintering process (**l**) Qualitative depiction of the stress–strain characteristics of uniform (black) and bilayer foams (red). The dashed red curves represent the tunable loading profile obtained at different layer thicknesses.

Hollow glass microspheres used here are from 10 to 100 μm in diameter (Figure 1a) and have high isostatic strength with low true density ranges (0.1–0.6 g/cc). These spheres come in contact with adjacent spheres in a mold. When the consolidated spheres are raised above their glass transition temperature (T_g_), they begin to flow in a viscous manner, leading to consolidation between adjacent spheres (Figure 1b). The consolidation via sintering process creates struts between spheres, resulting in a closed cellular structure with the increased strength (Figure 1c,d). Mechanical properties of glass foam are determined by the density and thus graded structures can be produced by layered foam structures with different densities. Gradient cellular structures are conventionally fabricated via gluing multiple cellular structures. Multi-layered glass foams can be sintered at the same time so the density change at the interface is not so abrupt. The interface promotes a smooth transition of energy absorption via sequential collapse and minimizes the peak stress at the impact by removing the effect of the adhesive at the interface. In order to achieve a layered structure, we developed a dry powder printing (DPP) system to fabricate bilayer foams with accurate control of layer thickness as well as achieving a consistent and uniform interface between the layers (Figure 1e–i). A consistent printing rate (Figure 1f), and uniform spatial printing were achieved (Figure 1h).

The bilayer structures were printed using two types of spheres with different wall thicknesses, and the printed powder compacts were sintered in a furnace (Figure 1j,k). Therefore, introducing different densities in a bilayer sample would enable tuning of the stress–strain curve by allowing sequential compression starting with the lower density layer, followed by the higher density layer. Figure 1l shows that the qualitative depiction of the stress–strain characteristics of uniform (black) and bilayer foams (red). The thickness ratio between the low and high density foam layers can be varied to represent the tunable loading profile. Three configurations of thickness ratios between layers were used. The sintered bilayer foam presents a sequential collapse of the constituent layers, manifesting as a step-like progression in the stress–strain profile and the onset strain for the second plateau stress can be controlled by adjusting the thickness ratio of the layers.

In this research, four types of microspheres were used to sinter different combinations of bilayer configurations. The influence of the density difference between the layers was investigated with the mechanical compression test and the interface property of the bilayers was investigated by comparing sintered bilayer foam and epoxy-bonded bilayer foam.

## 2. Materials and Methods

Dry powder printing (DPP) system: a commercially available 3D printer was modified to develop a DPP system. The built-in extruding system was replaced with a customized dry powder dispensing nozzle (Figure 1e,g). The dry powder dispensing nozzle was 10 mm in diameter with 15 small holes (1 mm in diameter) to dispense spheres. Two DC vibrational motors were attached to the middle of the dispenser and actuation was controlled with an Arduino by adjusting pulse width modulation (PWM). Figure 1f shows that the DPP dispenses spheres in a well-controlled matter by adjusting PWM values. A dispensing rate of 0.2 g/min was chosen to print spheres. After 5 min of initial dispensing, the dispensing rate was found to be stabilized so actual printing began after 5 min. The layer height of the printing process was optimized and the width of the printed example in Figure 1h is the same as the diameter of the dispenser. Stainless steel tubular molds (67 mm inner diameter and 150 mm in height) were installed on a stainless-steel plate (Figure 1i). Powder printing was optimized with respect to feeding rate, infill rate, infill pattern, and layer height.

Glass foam fabrication: All glass foam samples were prepared in tubular molds using the DPP system. Four different hollow soda lime-borosilicate hollow glass microsphere (HGM) types (K1, K20, S32 and K46, 3M St. Paul, MN, USA) served as the feedstock materials in the DPP fabrication of the cellular structures described herein. Uniform foam samples were prepared for each HGM type (K1, K20, S32 and K46). sintered bilayer foam samples (K1/K46, K20/K46, S32/K46) were printed with three thickness ratios (1:3, 1:1, and 3:1), respectively. Lastly, epoxy-bonded bilayer foams K20/K46 and S32/K46 with a 1:1 height ratio were prepared. Three samples were fabricated for each specific case and their average values of density were used. A box furnace (Lindburg/Blue M, ThermoFisher scientific) was used to remove moisture and to sinter the HGMs in the molds. The temperature of the furnace was ramped up to 150 °C and soaked for 120 min to drive off moisture from the sample. The temperature was then ramped to 600 °C and a sample was soaked for 180 min to ensure uniform thermal distribution. The temperature was then raised to a bonding temperature of 840 °C using a slow ramp rate (0.5 °C/min), followed by a bonding soak time of 20 min. After the bonding process, the temperature was reduced to 560 °C using a ramp rate (0.5 °C/min), followed by a 360 min of annealing process to relieve residual stress in the sintered foams. Finally, the temperature was reduced to room temperature using a ramp of 0.5 °C/min.

Mechanical testing: All tested samples were carefully trimmed into cylindrical samples (33 mm in diameter with 1.0 aspect ratio) for uniaxial compression testing. For the epoxy bonded sample, uniform cylindrical samples were prepared, and the foams were glued together with epoxy (Loctite E-90FL, Epoxy Adhesive) to make a bilayer foam. Nominally, 0.03 to 0.05 g of epoxy was smeared onto the interface to make bilayer samples and cured for 24 h. The width, height, and weight of each sample were measured to calculate the density of each sample. Lastly, the coating layer (Plasti-dip) was sprayed on all the samples to mitigate brittle fractures. Quasi-static uniaxial compression testing was performed using a load frame (MTS, Eden Prairie, MN, USA) at ≈10^−3^/s strain rate. Three samples were prepared and tested to get stress–strain curves. All the plots in this study are averaged. Frequently, stress–strain curves show that the stress change from elastic to plastic behavior is not distinctive, thus the offset yield method was used to determine the yield strength. Therein, a line is constructed parallel to the initial linear region of the stress–strain curve but offset by 0.2% from the origin. The 0.2% offset yield strength is the stress at which the constructed line intersects the stress–strain curve. Stress–strain plots are smoothed with the 50% percentile filtering method to remove short noise. Post-measurement analysis of the compression testing produced stress–strain curves which provide critical mechanical properties, such as energy absorption, the onset of densification, and yield strength to be obtained. To observe its microstructure, cellular solid samples were cleaved and observed in the SEM (TESCAN, VEGA-II). To check material crystallinity, X-ray diffraction (Bruker D8 Advance powder diffractometer) was performed. Phases were identified using the ICDD/PDF2 database.

## 3. Results

### 3.1. Dry Powder Printing

Fabrication of a bilayer foam with dried microspheres with controlled thickness and a well-defined interface requires a system for dispensing the spheres in a well-controlled manner. As described in the experimental section, a vibration-assisted DPP system was developed by modifying a commercial 3D printer. Additionally, the powder dispenser was designed to dispense spheres with spatial uniformity (Figure 1g,h) The dry powder dispenser is 10 mm in diameter with 15 small holes. Each nozzle diameter was 1 mm to avoid clogging or erratic flow. The nozzle diameter also provides a controllable dispensing rate (Figure 1f). The area of the printed spheres via the multiple small holes in the nozzle is similar to the OD of the nozzle. The nozzle moves and prints in a zig-zag infill pattern and the spheres were printed up to the edge of the mold without any empty space at the edge (Figure 1i). The printing rate of DPP and a printing layer height were used to determine with the dispensing rate. Nine stainless steel molds are placed in the DPP system. Nine samples of K46 were printed and sintered. The average weight of the prepared foams was 6.79 ± 0.31 g, indicating that the differences between samples were at most 9%. As the density of the foams was calculated with dimensions, the density of the foams is 0.37 ± 0.01 g/cc, indicating that the DPP system prints samples in a reproducible manner.

### 3.2. Uniform Foams

**Figure 2:**
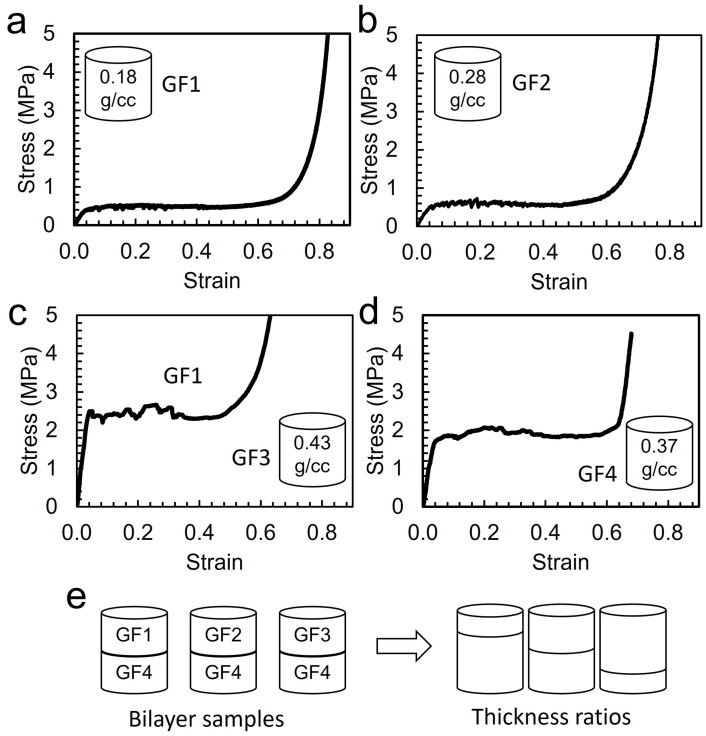
Stress–strain curves of (**a**) glass foam 1 (GF1) with 0.18 g/cc (**b**) GF2 with 0.28 g/cc (**c**) GF3 with 0.43 g/cc (**d**) GF4 with 0.37 g/cc. (**e**) configuration of bilayer glass foam using GF1-GF4 system and thickens ratios between the two layers.

Hollow spheres with different densities which are mainly determined by diameter and wall thickness are available. Uniform foams made with different spheres were tested in uniaxial compression tests and the stress–strain curves provide various information such as modulus, yield stress, plateau stress, and onset of densification strain. As the microspheres are sintered above the T_g_ for soda-lime-borosilicate glass, the walls of adjacent spheres start to form a strut, resulting in a foam with cellular microstructure. Four different hollow glass microspheres (HGMs) were used in the fabrication of the uniform glass foams. We name the foams as Glass Foam (GF1–GF4) for readers’ convenience. The densities of the foams are different from spheres’ true densities due to different degrees of consolidation at the sintering conditions. Foam density depends on volume shrinkage through sintering and the porosity of the foam. The density of uniform foam of four types of spheres used in this research is shown in Table 1. Given that the true density of GF2 spheres is 0.12 g/cc, the foam density of GF2 increased more compared to other foams through the sintering process. Each sphere has different consolidation kinetics with its density, wall thickness, and material which requires different optimal processing conditions. For example, at a given process temperature, GF3 spheres showed more shrinkage in terms of the diameter of the foam, resulting in a higher density of the foam. It is believed that the higher shrinkage of GF3 is due to different material composition of the feedstock spheres. To investigate the tunable properties of bilayer foams, identical processing parameters were applied for all the samples. Mechanical properties of the sintered foams were obtained using uniaxial compression testing. Figure 2 shows stress–strain curves of the four uniform foams. They all display three typical regimes: linear elastic region, plateau stress, and densification. At initial compression, the linear elastic regime starts via cell edge bending and cell face stretch. The energy under the elastic regime is proportional to the stiffness and thickness of the cell edge and wall, related to the relative density of the samples. The stress reaches yield stress, followed by long plateau stress which contributes to the energy-absorbing capacity of the cellular structures via the brittle collapse of the microstructure. After the brittle collapse, densification begins, the stress–strain curve rises steeply, and the foams with higher density exhibit an early onset of densification. The curves in Figure 2 show large differences in modulus, yield strength, plateau stress and the onset of densification between these foams. For achieving tunability, plateau stress is considered mainly. As shown in Figure 2, the plateau stresses are related to foam density. These stress values were used to characterize the bilayer foam. Three sets of bilayer foams (GF1/GF4, GF2/GF4 and GF3/GF4) were prepared with different thickness ratios (e.g., GF1:GF4 = 3:1, 1:1, and 1:3) (Figure 2e) to demonstrate tunable stress–strain profiles. XRD result shows the sintered foams are high crystallinity. The main phase is cristobalite SiO_2_. The compressive strength of the foam might be different with regard to the degree of the material’s crystallinity, but the brittle collapse of the cellular microstructure is the primary mechanism of energy absorption. Therefore, the study on the tunable stress–strain curves using glass foam still shows valid results.

### 3.3. Bilayer System

**Figure 3:**
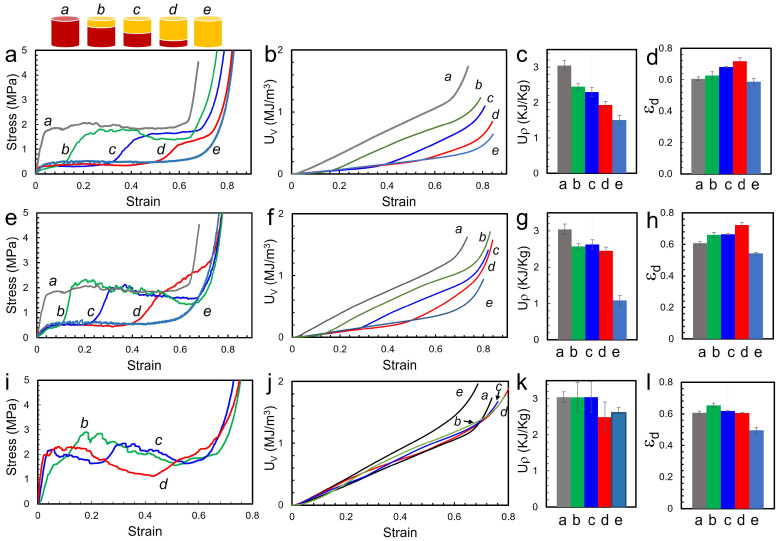
GF1 and GF4: (**a**) stress–strain curve (**b**) cumulative energy absorption, *U_V_* (**c**) specific energy absorption, *U_ρ_* (**d**) densification strain, ϵ_d_; GF2 and GF4: (**e**) Stress–strain curve (**f**) cumulative energy absorption, *U_V_* (**g**) specific energy absorption, *U_ρ_* (**h**) densification strain, ϵ_d_; GF3 and GF4: (**i**) Stress–strain curve (**j**) cumulative energy absorption, *U_V_* (**k**) specific energy absorption, *U_ρ_* (**l**) densification strain, ϵ_d_.

A series of bilayer foam samples were fabricated via the sintering process and they were subjected to quasi-static uniaxial compressive load with 10^−3^/s strain rate. Figure 3a shows the stress–strain profile of bilayer foams with three thickness ratios of GF1/GF4 and uniform foams of GF1 and GF4. Each curve of bilayer foams in the profile shows a distinctive two-step stress–strain profile due to its sequential collapse. The profile exhibited in the bilayer foams is similar to the conceptual depiction of the stress–strain profile shown in Figure 1l. The low-density GF1 layer is compressed first for all three cases and their plateau stress is slightly less than 0.5 MPa. The curves start to increase at the end of the first plateau and reach the second plateau with the onset of compression of the GF4 layer. The strain at which the stress rises to the second plateau stress differs with the ratio of layer thickness. The first plateau region ends early at about strain 0.1 with a thinner GF1 layer (curve (b)) and the first plateau ends around strain 0.5 for the foam with a thicker GF1 layer (curve (d)). The slope of the transition to the second plateau was found to be changing as the thickness ratio of the bilayers is configured differently. Curve (b) shows a much steeper transition and curve (d) shows the least steep transition. Curve (d) has a 56% difference in modulus from curve (b) and the modulus of curve the (c) is located in between (b) and (d). The second plateau stress values are different with the configurations of the layer thickness. The curve (b) with a thicker GF4 layer shows a larger 1.7 MPa while the curve (d) with a thinner GF4 layer is about 1.2 MPa. These values are lower than the one of uniform GF4 (curve (a)) ( 2 MPa). The interface region of the sintered bilayer foams usually includes large voids clusters due to induced interfacial stress from the different shrinkage rates of the sphere types. Lastly, the foams start to densify, and their onset can be shifted, which appears to be related to the GF1 layer thickness. The curve (d) with a thicker GF1 layer shows the densification strain 14% higher compared to the curve (b) with a thin GF1 layer. The onset of densification strain and the transition slope between two plateau stresses can potentially be tunable value that can be designed to reduce injury. Cumulative energy absorption per unit volume (*U_V_*) (Equation (1)) of both uniform and bilayer foams is shown as a function of strain in Figure 3b and the figure is calculated simply from the area under the stress–strain curves. The figure clearly shows uniform GF1 (e) and GF4 foams (a) deliver minimum and maximum energy absorption. Bilayer foams are located in between the uniform foams. The foam with a thicker GF4 layer is closely located to the uniform GF4 as expected. The bar graphs in Figure 3c show specific energy absorption (*U_ρ_*) for each foam. *U_ρ_* was obtained by dividing *U_V_* with the density of the foam (Equation (2)). The graph also shows gradual changes with varying layer thickness ratios of the two layers. Figure 3d shows ϵ_d_ which is the densification strain, indicating that bilayer foam contributes to the increase in ϵ_d_. The onset of densification (ϵ_d_) was determined from the energy absorption efficiency plot. Energy absorption efficiency is defined as the energy absorbed at a given applied stress to the sample [40] (Equation (3)). The maximum of the efficiency (*e*_max_) is determined where *de/dϵ* = 0 and ϵ_d_ corresponds to the strain level at *e*_max_:

(1) $$U_{V}=\int_{0}^{\epsilon_{d}}\sigma \;d\epsilon \;$$

(2) $$U_{\rho }=\frac{U_{V}}{\rho }$$

(3) $$e\left(\epsilon \right)=\frac{1}{\sigma (\epsilon )}\int_{0}^{\epsilon_{d}}\sigma \left(\epsilon \right)\;d\epsilon \;$$

Figure 3e shows the stress–strain plot of bilayer foam GF2/GF4. The density of the sintered uniform foam is 0.28 and 0.37 g/cc for GF2 and GF4, respectively. The curves are very similar to GF1/GF4 in Figure 3a. Moduli in the linear elastic region are 56 and 24 MPa for uniform GF4 and GF2 (curve a and e) respectively via cell edge bending and cell face stretch. After the long plateau stress via brittle collapses, densification occurs, and the stress–strain curve rises steeply. Denser GF4 exhibits densification more abruptly while GF2 shows slow and long densification. Bilayer GF2/GF4 is similarly showing the two-step stress–strain profile. The thickness ratio of the layers also determines the strain where the second plateau stress starts. The transition from first to second plateau stress is smoother for curve (d) which is thinner GF4 and the difference in the slope of the transition is about 120%. The plateau stress of curve (b) and (c) show a gradual decrease which can be explained by the brittle nature of the foam which was not observed in GF1/GF4 bilayer foam since the density of sintered GF2 foam is higher than GF1 foam. While the curve (b) and (c) show a decreasing trend, curve (d) shows an increase in the second plateau stress, and the increase can be explained by a less severe fracture of the thinner GF4 layer and the combination of plateau stress and densification process. The thicker GF4 layer would have a higher chance to be fractured since the uniaxial compression would propagate via the thicker layer. The onset of densification of the bilayer foam is increased up to 20% from uniform foam. Cumulative energy absorption (*U_V_*) of the GF2/GF4 bilayer is shown as a function of strain in Figure 3f and demonstrates tunability. Specific energy absorption (*U_ρ_*) in Figure 3g shows that the (*U_ρ_*) values of bilayer foams are in between the uniform foams. The onset of densification (ϵ_d_) in Figure 3h shows that the bilayer foams have larger ϵ_d_ values than uniform foams.

Lastly, GF3/GF4 bilayer foams were analyzed Figure 3i–l. Uniform GF3 and GF4 foams have similar density values of 0.43 and 0.37 g/cc, respectively. Figure 3i shows the mechanical response of GF3/GF4 bilayer foams. The curves for the uniform foams were omitted from Figure 3i for clarity. Uniform GF3 foam overall provides slightly higher mechanical properties than GF4 foam with higher modulus, plateau stress but the onset of densification is at a lower strain of 0.5. (Figure 2) Bilayer foams of GF3/GF4 are similarly showing the two-step stress–strain profile like other bilayer foams. The strain for the onset of second plateau stress is located with changing thickness ratio of the layers. A gradual decrease in plateau stress becomes more significant due to the brittleness of the denser foams. Additionally, interfacial stress could have induced additional fracture that promotes a gradually decreasing trend of plateau stress (curve b, c and d). The transition from first to second plateau stress is smoother for curve (d) which is thinner GF4. Cumulative energy absorption of GF3/GF4 bilayer in Figure 3j plot does not display a clear tunable property. Most of the energy absorption lines overlapped over the measured strain range. The *U_ρ_* in Figure 3k show that the bilayer sample (b) and (c) has similar specific energy absorption to uniform GF3. The stress–strain curve shows that GF4 initially gets crushed providing first lower plateau stress and it is followed by GF3 crush at higher plateau stress. Even though the tunable energy absorption was not achieved, the stress–strain profile can be still tunable, so the initial impact acceleration can be reduced in the first plateaus stress, and still impact energy can be still absorbed by the second plateau stress. The onset of densification of bilayer foam is increased above the 0.61 strain range which is larger than the uniform foams (Figure 3l). The increase in onset of densification is observed on all three sets of bilayer samples, including GF1/GF4, and GF2/GF4.

### 3.4. Epoxy-Bonded vs. Sintered Bilayer Foams

**Figure 4:**
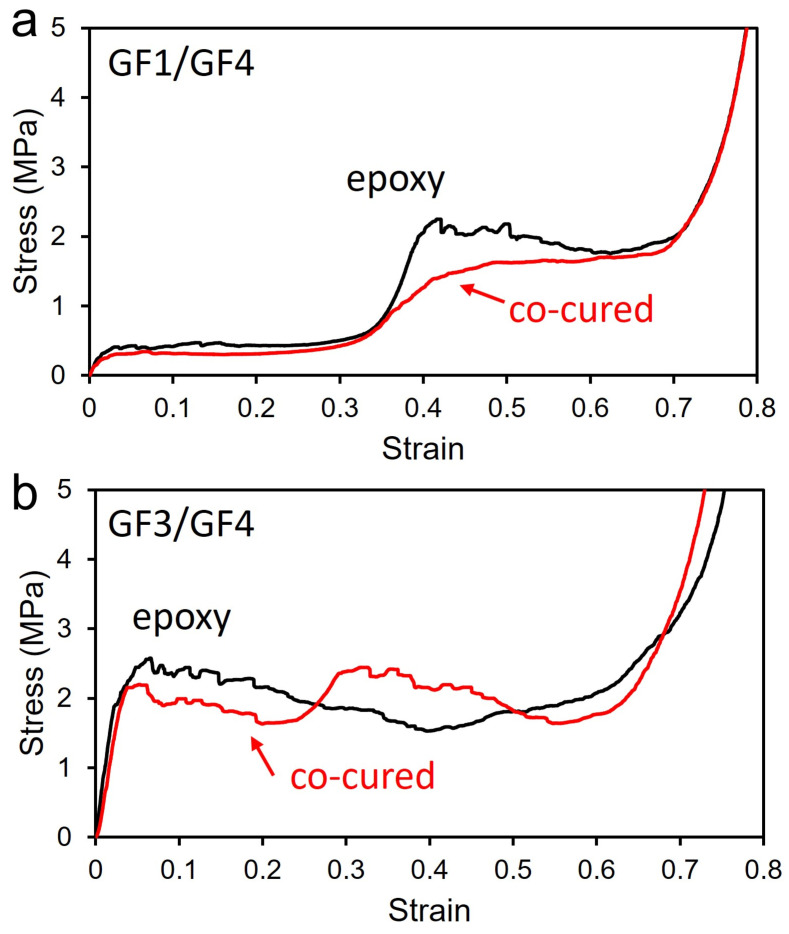
(**a**) Stress–strain curves of sintered bilayer foam of GF1/GF4 (red line), and epoxy-bonded bilayer foam (black line). (**b**) Stress–strain curves of sintered bilayer foam of GF3/GF4 (red line), and epoxy-bonded bilayer foam (black line).

Figure 4a shows the compression tests of the epoxy-bonded samples. The foam behaves differently with their stress rising with a slightly higher modulus than their sintered counterparts. The modulus increase in the epoxy-bonded foams is 75% for GF1/GF4. Stress–strain curves of sintered and epoxy-bonded GF1/GF4 foams are similar up to a strain of about 0.35. As the second layer, which is GF4, starts to get compressed, stress increases up to 1.7 MPa in both foams. Epoxy-bonded foam exhibits a large overshoot of stress up to 2.3 MPa and comes down to 1.9 MPa gradually. On the other hand, sintered foam shows a much smoother transition. This difference in transition may play an important role in terms of injury. Plateau stress can preferably be just below that which would cause damage to the protected object while absorbing maximal energy. Therefore, the steep increase and overshoot of the force could put more stress on a passenger, and thus the smoother transition is preferred. Densification begins around 0.68 for both sintered and epoxy GF1/GF4 foams. The energy absorption of epoxy-bonded foam is 19% larger than sintered foam. Epoxy foam made of GF3/GF4 shows different mechanical responses from the epoxy foam of GF1/GF4 (Figure 4b). Modulus increase in the epoxy-bonded foams is 11% for GF3/GF4. Sintered GF3/GF4 foam shows a distinctive two-step compression behavior of bilayer structures. As stress increases steeply in the elastic regime, it starts to manifest plateau stress up to 0.25 strain. Stress increases again up to 2.5 MPa and plateau stress with gradual decrease exists until the onset of foam densification at 0.62 strain. On the other hand, the epoxy-bonded foam has higher peak stress, followed by long plateau stress without second peak stress up to 0.6 strain which is the onset of densification. Since the density of the two materials is similar, the epoxy-bonded bilayer shows the compression behavior of uniform foams. However, sintered bilayer foam has a unique interfacial structure containing larger void clusters. The void clusters were formed during sintering due to the different shrinkage rates of the HGM. The void cluster in the interfacial region leads to initial compression in GF3 layer first, followed by compression of GF4 layer. A more in-depth study on the interfacial void cluster of bilayers was published previously [24]. In the GF1/GF4 system, the foams have a large difference in their densities, so the effect of the interfacial void cluster is less significant. The average energy absorption of sintered samples (3.05 kJ/kg) is slightly higher than that of the epoxy-bonded sample (2.74 kJ/kg) for GF3/GF4 system.

### 3.5. Energy Absorption and Densification Strain

**Figure 5:**
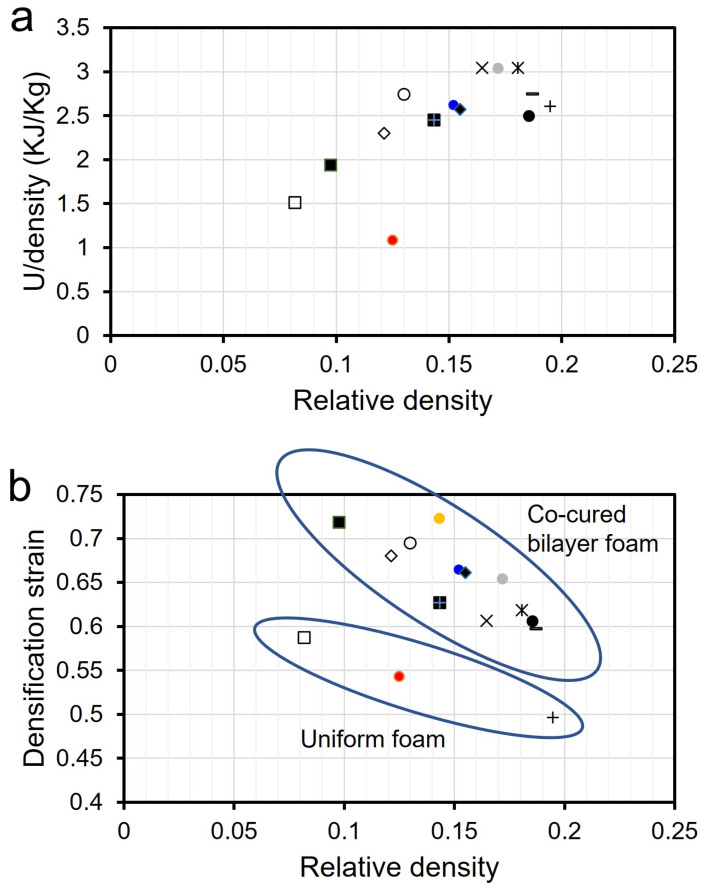
(**a**) Specific energy absorption (*U_ρ_*) of uniform and bilayer foams as a function of relative density. (**b**) densification strain (ϵ_d_) of uniform and bilayer foams as a function of relative density.

Specific energy absorption (*U_ρ_*) of all the tested samples, including uniform foams and bilayer foams, is plotted as a function of the relative density of foams in Figure 5a. Relative density (ρ/ρ_s_) was obtained by dividing the density of the foam with a bulk density of 2.23 g/cc (ρ_s_). Specific energy absorption increases with an increasing relative density of foams. As shown in Figure 5a, sintering bilayer foams enable them to produce a broad range of densities of glass foams with fine density control. Even finer density controls would potentially be possible by multiple layers in conjunction with the printing of the DPP capability. On the other hand, density control of uniform foams can be done by adjusting sintering temperature or duration which is more challenging. An increase in *U_ρ_* in a linear manner as a function of relative density demonstrated that tunable energy absorption can be achieved via a sintered bilayer approach.

**Figure 6:**
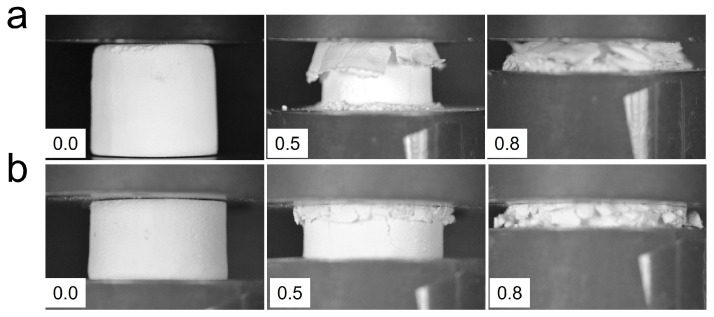
Images of compression testing of (**a**) sintered bilayer foam GF3/GF4 and (**b**) GF3 uniform foam. The numbers in inset are strain.

Densification strain values of all the samples are shown in Figure 5b. Densification strain increases with the decrease in relative density and interestingly, there is a clear trend that the bilayer system has a higher densification strain than uniform foam. Both bilayer and uniform foam cases show that densification strain appears at lower strain with larger relative density since a denser foam begins densification earlier. In the densification regime, the individual cell walls or struts come into contact with one another and provide drastically increased stiffness. In bilayer foams, the distinctive sequential collapse, such as a lower to higher density, allows separation between the neighboring cellular struts in the foam, leading to later densification. Figure 6 shows images of the bilayer and uniform glass foams that were captured during compression testing. Bilayer foam in Figure 6a started to compress from the upper layer, and then the side walls of the compressed layer spalled significantly in the lateral direction at 50% strain. On the other hand, uniform foam in Figure 6b indicates that it is compressed from the top but it does not show lateral sidewall spalling as bilayer foam showed, indicating the compressed layer of the uniform foam is accumulated vertically instead of being spalled outward. The vertical accumulation during compression can be the reason for the early densification of uniform foam. Spalling outward of the first layer of bilayer helps delay the onset of densification.

## 4. Conclusions

In summary, several types of hollow glass spheres were sintered to produce bilayer cellular structures. The mechanical properties of all three bilayer configurations produced by sintering show tunable strain–stress profiles via controlling layer thickness ratios. The smoothness of transition from first to second plateau stress was found to be tunable. Overall, the onset of densification of the bilayer is determined by the foam with lower density, resulting in delayed onset of densification. Energy absorption was found to be tunable when the difference in densities of the two foams is greater (GF1/GF4 and GF2/GF4). When the difference is small, energy absorption was found to be not tunable but overall absorption was greater than uniform foams with help of delayed onset of densification (GF3/GF4). The mechanical properties of bilayer foams fabricated by sintering and epoxy bonding were compared. The bilayer foams with a larger density difference (GF1/GF4) showed the same two-step stress–strain profile except that the smooth transition between the first and second plateau stress is more abrupt and exhibits a higher peak in epoxy-bonded foam. The bilayer foams with smaller density differences (GF3/GF4) show different profiles and the epoxy-bonded foam did not show two-step profile. The critical feature of the sintered bilayer foam is the tunability that would be achieved by programming the thickness of each layer so that the range of each plateau stress can be adjusted, allowing the control of energy absorption. Eventually, this capability exhibited by the sintered bilayer can limit impact stress on an occupant and perform effective energy absorption to minimize injury. Designing multilayer foam by increasing the number of layers can allow finer tunability and gradual increase in stress to be achieved for diverse impact conditions. 

## Figures and Tables

**Table 1:** Glass foam used in the experiments.

Name	3M Name	Foam Density (g/cc)	Sphere Density (g/cc)	Sphere Diameter (μm)	Wall Thickness (μm)
GF1	K20	0.18	0.20	60	0.93
GF2	K1	0.28	0.12	65	0.62
GF3	S32	0.43	0.32	40	1.01
GF4	K46	0.37	0.46	40	1.48

## Data Availability

The data presented in this study are available on request from the corresponding author.

## Abbreviations

The following abbreviations are used in this manuscript:

DPP	Dry Powder Printing
GF	Glass Foam
HGM	Hollow Glass Microsphere
PWM	Pulsed Width Modulation
SEM	Scanning Electron Microscopy
